# TLR9 Signaling Suppresses the Canonical Plasma Cell Differentiation Program in Follicular B Cells

**DOI:** 10.3389/fimmu.2018.02281

**Published:** 2018-11-28

**Authors:** Bárbara José Antunes Baptista, Alessandra Granato, Fábio B. Canto, Fabricio Montalvão, Lucas Tostes, Herbert L. de Matos Guedes, Antonio Coutinho, Maria Bellio, Andre M. Vale, Alberto Nobrega

**Affiliations:** ^1^Department of Immunology, Institute of Microbiology, Federal University of Rio de Janeiro, Rio de Janeiro, Brazil; ^2^Institute of Biophysics Carlos Chagas Filho, Federal University of Rio de Janeiro, Rio de Janeiro, Brazil; ^3^Instituto Gulbenkian de Ciência, Oeiras, Portugal

**Keywords:** follicular B cell, plasma cell, TLR9, TLR4, limiting dilution

## Abstract

The relative potency and quality of mouse B cell response to Toll-like receptors (TLRs) signaling varies significantly depending on the B cell subset and on the TLR member being engaged. Although it has been shown that marginal zone cells respond faster than follicular (FO) splenic B cells to TLR4 stimulus, FO B cells retain full capacity to proliferate and generate plasmablasts and plasma cells (PBs/PCs) with 2–3 days delayed kinetics. It is not clear whether this scenario could be extended to other members of the TLR family. Here, using quantitative cell culture conditions optimized for B cell growth and differentiation, we show that TLR9 signaling by CpG, while promoting vigorous proliferation, completely fails to induce differentiation of FO B cells into PBs/PCs. Little or absent Ig secretion following TLR9 stimulus was accompanied by lack of expression of cell surface markers and canonical transcription factors involved in PB/PC differentiation. Moreover, not only TLR9 did not induce plasmocyte differentiation, but it also strongly inhibited the massive PB/PC differentiation of FO B cells triggered by LPS/TLR4. Our study reveals unexpected opposite roles for TLR4 and TLR9 in the control of plasma cell differentiation program and disagrees with previous conclusions obtained in high-density cultures conditions on the generation of plasmocytes by TRL9 signaling. The potential implications of these findings on the role of TLR9 in controlling self-tolerance, clonal sizes and regulation of humoral responses are discussed.

## Introduction

Toll-like receptors (TLRs) are a family of structurally related proteins highly conserved in evolution and consist of at least 11 members in mammals. They recognize a distinct set of also highly conserved molecules of microbial origin, such as LPS (TLR4), lipoteichoic acids (TLR2), CpG-rich DNA (TLR9), flagellin (TLR5), viral ssRNA (TLR7/8) (1, 2). TLRs play a critical role in innate and adaptive immune response to microorganisms and are expressed in all hematopoietic derived cell types involved in immunity (3–7). Beside their critical role in myeloid cell types, the direct action of TLRs on T and B cells are increasingly appreciated because of their immune-regulatory potential, controlling the quality of TCR and BCR signaling, with important implications for immune responses to microbiota and autoimmunity (8–15).

Mouse B cell activation with lipopolysacharide (LPS), the canonical TLR4 ligand, has long been characterized and the genetic control of B cell mitogenic response to LPS played an instrumental role in the cloning and identification of TLR4 (16–19). LPS is not only a potent stimulus for mouse B cell proliferation, but also for the generation of plasmablasts/plasma cells (PBs/PCs), being a model of choice for studying plasmocyte differentiation, *in vitro* and *in vivo* (20, 21). B cell response to LPS/TLR4 stimulus varies in quantity and quality among the different B cell subsets. *In vitro* studies showed that marginal zone (MZ) B cells proliferate and generate (PBs/PCs) with a faster kinetics when compared to follicular (FO) B cells (22, 23). Importantly, when stimulated with saturating doses of LPS, FO B cells abundantly proliferate and generate PBs/PCs with a 2–3 days delay in kinetics when compared to MZ B cells; at lower, suboptimal doses, only MZ cells respond efficiently to LPS stimulus (17, 22, 23).

More recently, the B cell response to TLR signaling other than TLR4 has been addressed (24, 25). Again it has been observed that the relative potency of the B cell response to different TLR stimuli varies very significantly depending on the B cell subset; substantial differences were also noted depending on which TLR member was engaged, although the reasons for that are not clearly understood. Differential responses to distinct TLR ligands could simply reflect different levels of receptors expressions, but could also be related to differences between their signaling pathways. Interestingly, crosstalk between multiple TLR signaling pathways, with higher or lower responses, have been shown to alter B cell activation and effector functions, including class-switch recombination (CSR) (26).

It is established that FO B cells retain the full capacity to proliferate and massively generate PBs in response to LPS, both in frequency and magnitude (17, 22). Less clear, however, if this is also the case for other TLR ligands. Published studies show that FO B cells proliferate vigorously to TLR1/2, TLR2/6, TLR7, and TLR9 agonists, but the data indicating whether substantial generation of PBs/PCs by these stimuli could occur with delayed kinetics, as for LPS, are less clear. It has been reported that FO B cells respond less well than MZ B to TLRs stimuli, the magnitude of the Ig secretory response typically varying 10-fold when comparing both populations. However, it is difficult to interpret the significance of these findings as these experiments were all done in high-density cultures conditions, more than 0.5 −1.0 × 10^6^ cells/ml, where proliferation, overgrowth, death and differentiation may balance each other and a few percent of responding cells may overtake the culture. Although the amount of secreted Igs measured in culture supernatants of FO B cells is diminished, the frequencies of growing B cells clones that differentiate into PBs/PCs have not been determined. Thus it is not possible to ascertain whether the reduced amount of Ig is due to delayed kinetics of PB generation, to a general defect in PB differentiation or to a lower frequency of fully responding FO B cells. Of special interest is the proper estimation of the frequency of FO B cells that fully differentiate into PBs/PCs under TLR9 stimulus, because of the suggested role of TLR9 signaling on the breaking of tolerance and autoimmunity (27); whether this is a rare event or a more common feature remains to be properly established.

Here, using a limiting dilution assay (LDA) strategy and non-saturating optimal cell culture conditions, we compare TLR4 and TLR9 agonists in promoting proliferation and plasmocyte differentiation of follicular (FO) splenic B cells, as measured by responding cell frequencies, Ig secretion, levels of expression of cell surface markers (CD138, B220) and PB canonical transcription factors (IRF4, BLIMP1, PAX5, and XBP1/s). Of note, we found that TLR9 signaling totally fails to induce plasmocyte differentiation of FO B cells. Accordingly, the frequency of PBs/PCs detected in LDA was none or minimal (<1/1000); the expression of CD138 was profoundly reduced and transcription factors involved in plasmocyte differentiation were poorly induced by CpG under optimal cultures conditions, indicating a general defect in PB differentiation. Importantly, not only TLR9 signaling did not induce plasmocyte differentiation, but it also strongly inhibited PB/PC differentiation in FO B cell cultures stimulated with LPS. The present study brings evidence against the currently accepted view on the capacity of TLR9 signaling to promote plasmocyte differentiation of FO B cells and discloses unexpected opposite roles for TLR9 and TLR4 in the control of the canonical plasma cell differentiation program.

## Materials and methods

### Mice and cells

C57BL/6, 8–10 weeks of age, were obtained from animal facilities of Federal University of Rio de Janeiro, UFRJ and Federal Fluminense University, UFF. TLR9 KO mice Tlr9–/– mice were donated by Dr. S. Akira (Osaka University, Japan) and bred in the LAT animal facility of Federal University of Rio de Janeiro, UFRJ. Experimental procedures were approved by “Comitê de Ética do Centro de Ciências da Saúde CEUA – CCS/UFRJ”. Spleen cell suspensions were obtained by gently teasing spleens onto a cell strainer with ice-cooled complete medium (OptiMEM supplemented with 10% FBS, 5 × 10^−5^ M 2-βME, streptomycin 100 μg/ml, penicillin 100 U/ml) (Life Technologies, Grand Island, NY). Erythrocytes were depleted using ACK buffer (0.155 M NH_4_Cl, 10 mM KHCO_3_, 0.1 mM sodium EDTA). Cells were counted using a hemocytometer with exclusion of dead cells with trypan blue dye.

### Cell staining, flow cytometry and cell sorting

Antibodies used for surface staining were PE anti-CD23 (B3B4), FITC anti-CD21 (7G6) (BD PharMingen, San Diego, CA); PECy7 anti-CD23 (B3B4), PECy7 anti-B220 (RA3-6B2), PECy7 anti-CD21 (7G6), APC anti-CD93 (AA4.1) (eBioscience, San Diego, CA), PE anti-CD138 (Biolegend, San Diego, CA), and DyLight® 649 goat F(ab) fragments anti-mouse IgM (Jackson Laboratories, Bar Harbor, ME). Cells were incubated with Abs in FACS buffer (PBS, 5% FBS, 0.05% Na-azide) for 20 min at 4°C and washed with FACS buffer. For intracellular staining, cells were fixed and permeabilized after surface staining using Foxp3 transcription factor staining buffer set protocol and reagents from eBioscience. Antibodies with the following specificities were used for intracellular staining: Alexa Fluor 488 anti-PAX5, Alexa-647 anti-BLIMP1, Pe-Cy7 anti-IRF4, all from BioLegend. In non-fixed samples, propidium iodide was added at 0.5 μg/ml immediately before data acquisition, for dead cell exclusion. Dead cell exclusion in fixed cell samples was done using the Zombie Red™ Fixable Viability kit (BioLegend). Data were acquired by a FACSCalibur™ (BD Biosciences, San Jose, CA) and analyzed using CELLQuest™ software (BD Biosciences) or Summit™ (DakoCytomation, Glostrup, Denmark). MoFlo™ flow cytometer (DakoCytomation) was used for sorting of splenic B cells (B220+), splenic follicular B cells (FO B) (CD93-B220+CD21lowCD23+), splenic marginal zone B cells (MZ B) (CD93-B220+CD21highCD23-). After cell sorting, FO B cell populations showed purity higher than 99 %.

### CFSE staining

For cell proliferation analysis, cells were stained with CFSE (Carboxyfluorescein diacetate succinimidyl ester) immediately prior to the culture. Briefly, sorted B cells were incubated at 5 × 10^6^ cells/ml with 0.5 μM CFDA SE (Molecular Probes) in pre-warmed PBS for 5 min at 37°C and washed twice with complete OptiMEM medium.

### ^3^H-thymidine incorporation

3Htdr uptake: 18 h before harvesting, 1 μCi of tritiated thymidine (4.0 Ci,mmoLSIGMA-ALDRICH,) was added to each well. The cells were harvested on glass wool filters and the radioactivity incorporated in the DNA was measuredby liquid scintillation spectrometry. Results are expressed as the mean value of cpm from triplicate cultures.

### B cell cultures

FACS-sorted FO B cell populations, >99% purity, were cultured in 96-well flat-bottom plates (Corning Incorporated, NY) at varying cell densities/well, 200 μl/well, in RPMI 1640 supplemented with 10% FCS, 5 × 10^−5^ M 2-β-ME, streptomycin 100 μg/ml, penicillin 100 U/ml (Life Technologies) in humidified atmosphere of 5% CO_2_ at 37°C. Sorted B cells were cultured upon a monolayer of irradiated S17 stromal cell line (28); briefly, one day before adding B cells to cultures, plates were seeded with 3 × 10^3^ S17 per well, 100 μl/well, and incubated overnight at 37°C with 5% CO2. The next day, the S17 culture plates were irradiated with 2,000 rads and received the B cells, 100 μl/well. B cells were stimulated with LPS or CpG; LPS from *E. coli*055:B5 strain, CpG ODN 1826 and 1668 (both from InvivoGen, San Diego, CA). Proliferation was measured either by tritiated thymidine uptake, or CFSE dilution, as above. Recovered live cells were counted using hemocytometer with exclusion of dead cells with trypan blue dye, and processed for flow cytometric or quantitative RT-PCR analysis. Statistical analysis (unpaired Student's *t*-test and ANOVA with Bonferroni post-test) were done using Prism^®;^ software (GraphPad Software, Inc., San Diego, CA, USA); results were considered statistically significant if *p* < 0.05.

### Quantitative RT-PCR analysis

RNA was extracted using an RNeasy Micro kit (Qiagen, Düsseldorf, Germany) according to the manufacturer's instructions. Total cDNA was prepared using random primers (Promega, Madison, WI) and Improm-II reverse transcription system (Promega, Madison, WI) following manufacturer's instructions. Quantitative PCR reactions were performed in duplicate with Power SYBR Green PCR Master Mix (Applied Biosystems, Foster City, CA), and detection was done using an Step-One Real-Time PCR system (Applied Biosystems, Foster City, CA) and normalized to the amount of housekeeping gene 18S rRNA. Reactions were incubated at 95°C for 10 min and then run through 45 cycles of 95°C for 15s and 60°C for 1 min. For quantification of expression, the following primers were used:

Pax-5: R: 5′- GTCTCGGCCTGTGACAATAGGGTAG-3′
  F: 5′-CGCGTGTTTGAGAGACAGCACTACT−3′
Blimp-1: R: 5′-AGTGTAGACTTCACCGATGAGG-3′
  F: 5′-GAACCTGCTTTTCAAGTATGCTG-3′
XBP-1/s: R: 5′-CCAGAATGCCCAAAAGGATA-3′
  F: 5′-GGAGTGGAGTAAGGCTGGTG-3′
18SrRNA: R: 5′-CGCGGACACGAAGGCCCCAAA-3′
  F: 5′-GGGCGTGGGGCGGAGATATGC-3′

### Limiting dilution assay (LDA)

varying numbers of B cells were cultured in 250 μl of complete RPMI 1640 medium in 96 well flat-bottom upon a monolayer of growth-supporting S17 cells, 3 × 10^3^/well (21).The preparation of S17 as feeder cell was done as follows: briefly, one day before start of LDA cultures, plates were coated with 3 × 10^3^ S17 per well and incubated overnight at 37°C with 5% CO_2_. The next day, the S17 culture plates were irradiated at 2,000 rads and received the B cells at variable numbers in 44 replicates for each cell concentration, averaging 32; 16; 8; 4; 2;1; 0.5; and 0.25 B cells per well, to determine the frequency of IgM secreting clones by ELISA according to Poisson's distribution (29, 30). Culture supernatants were harvested on day 7 of culture.

### ELISA and ELISPOT

To measure IgM or total Ig in the culture supernatants, ELISA was performed using anti mouse IgM-specific or anti-mouse Ig reagents (Southern Biotechnology). Briefly, microplates (Costar, 96 well plate, #3690) were coated overnight at 4°C with 2.0 μg/ml of anti-mouse IgM or anti-mouse Ig, in PBS. The wells were washed with PBS and blocked with PBS 1% BSA (SIGMA-ALDRICH) for 2 h at room temperature. Samples were diluted in PBS-1%BSA-0.1% Tween20 (SIGMA-ALDRICH) and incubated overnight at 4°C. The wells were then washed with PBS. Secondary antibody was diluted in PBS-1% BSA-0.1% Tween, and added for 2 h at room temperature. The wells were further washed with PBS and the reaction revealed with OPD substrate (SIGMAFAST P9187). The reaction was stopped with 50 μl of HCl 1N and read at 490 nm. For ELISPOT, ELISA plates were coated with anti-Ig (Southern Biotechnology, Birmingham, AL), blocked with gelatin, and the cells were seeded according to a serial dilution and incubated at 37°C for 4–5 h. After washing out the cells, the spots, generated by the specific binding of secreted Ig, were revealed with peroxidase-conjugated anti-mouse Ig (Southern Biotechnology Associates, Birmingham, AL) and the reaction was developed using a commercial AEC substrate kit (BD ELISPOT).

## Results

**Figure 1:**
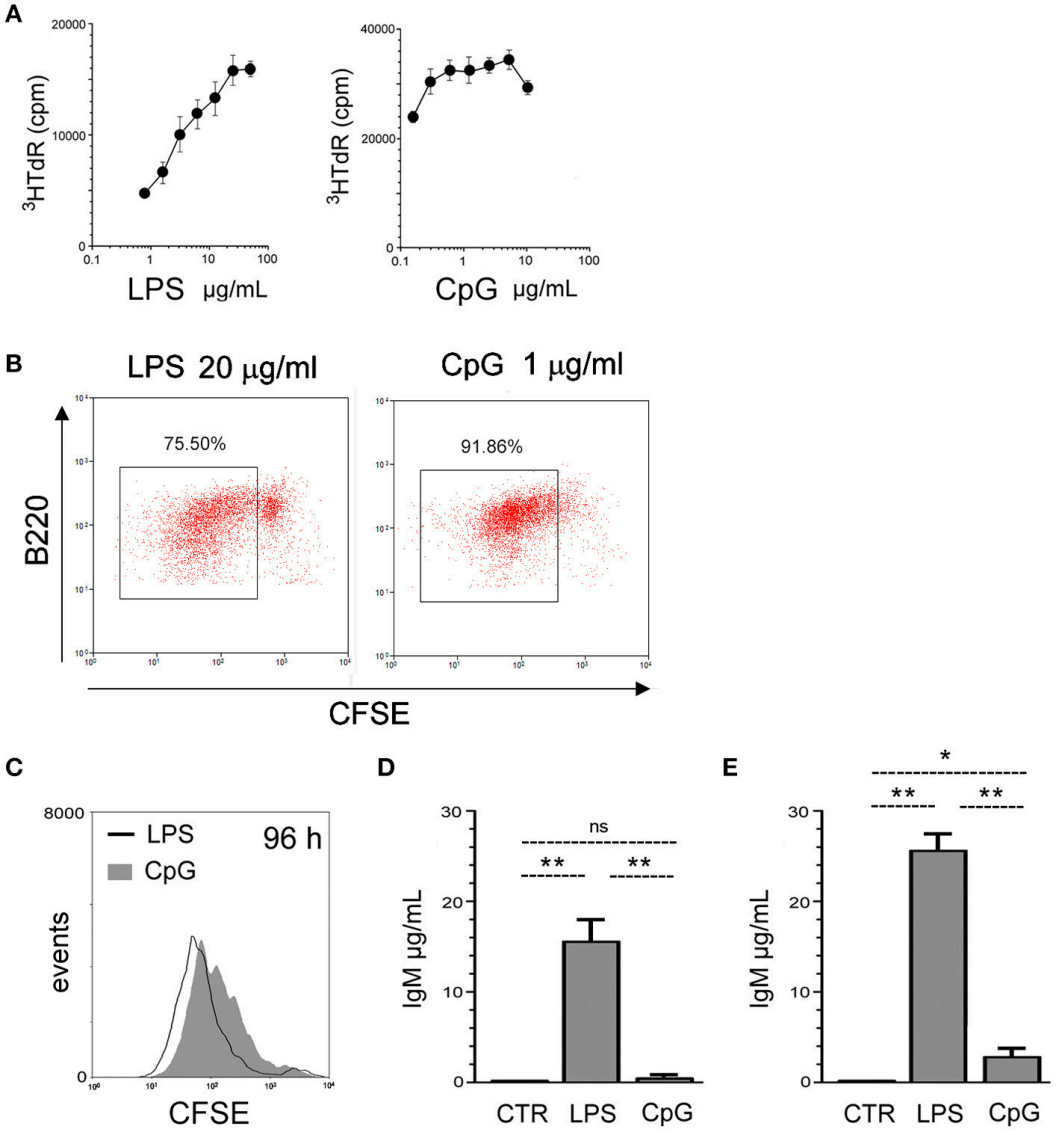
FO B cell responses to TLR4 and TLR9 agonists. **(A)** 2 × 10^5^ spleen cells/well were cultured for 48 h, in the presence of varying doses of LPS (left) or CpG (right); proliferation was measured by tritiaded thymidine incorporation (^3^HTdr cpm) in the last 16 h of culture;; graph shows mean ± SEM results from triplicates cultures of one experiment; two independent experiments were done with similar results. **(B)** 2 × 10^5^ spleen cells/well were loaded with CFSE and cultured for 72 h in the presence of LPS 20 μg/ml (left), CpG 1 μg/ml (right); proliferation of B220+ cells was evaluated by CFSE dilution; gated events shows cells that have divided. Dot-plot shows the result of one experiment; two independent experiments were done with similar results. **(C)** Splenic FO B cells, 2 × 10^5^ cells/well, were loaded with CFSE and cultured for 96 h in the presence of LPS 20 μg/ml or CpG 1 μg/ml; proliferation was evaluated by CFSE dilution. Histogram shows the result of one experiment; two independent experiments were done with similar results. **(D)** Splenic FO B cells, 2 × 10^5^ cells/well, were cultured for 7 days in the presence of LPS 20 μg/ml or CpG 1 μg/ml or left in medium alone (CTR); the supernatants were harvested and the amount of secreted IgM was measured by ELISA; graph shows mean ± SEM results from one experiment, with triplicates cultures for each group; results were considered statistically different if *p* < 0.05 Student's *t*-test (^*^); *p* < 0.01 (^**^); (ns), not significant.; three independent experiments were done with similar results. **(E)** Splenic B cells, 2 × 10^5^ cells/well, were cultured for 7 days in the presence of LPS 20 μg/ml or CpG 1 μg/ml or left in medium alone (CTR); the supernatants were harvested and the amount of secreted IgM was measured by ELISA; graph shows mean ± SEM results from triplicates cultures for each group; results were considered statistically different if *p* < 0.05, Student's *t*-test (^*^); *p* < 0.01 (^**^); (ns), not significant; two independent experiments were done with similar results.

We first performed B cell dose-effect proliferation assays in response to purified LPS and CpG (Invivogen), to characterize the saturation doses for both agonists; this is important as many controversial interpretations of data from B cell studies are due to comparisons of results obtained with sub-optimal agonist stimuli in some studies vs. optimal doses in others. Using ^3^HTdR incorporation assay in splenic cell cultures, we established here the doses of 20 μg/mL of LPS, and 1 μg/mL of CpG, as optimum doses for B cell proliferation (Figure 1A). Estimation of the number of mitotic cycles by CFSE dilution showed that CpG was more efficient than LPS in promoting the earlier entrance into cell cycle of a great majority of B cells, as measured at 72 h of culture (Figure 1B), coherent with the higher ^3^HTdR uptake at this time point. Sorted FO B cells (sorting gates in Supplementary Figure 1) proliferate as vigorously as total B cells in the presence of LPS or CpG (Figure 1C). At later time point, 96 h, LPS stimulated FO B cells exhibit higher percentage of cells with more mitotic cycles, whereas CpG stimulated B cells present less intense mitotic activity, as previously observed for total B cells (31).

FO B cells responded to saturating LPS stimuli with abundant IgM secretion in 7-days cultures (Figure 1D), as reported (22, 23); accordingly, FO B cells produced around 15 μg/ml of IgM, in 7-days high-density B cell culture with 2 × 10^5^ cells/well. However, the amount of IgM in the supernatant of CpG-stimulated FO B cells was 2 logs lower, around 0.2 μg/ml; for comparison, the results obtained with non-sorted, total splenic B cells are shown (Figure 1E). The lower amount of IgM measured in the supernatants of CpG-stimulated FO B cell cultures could reflect a lower frequency of PB generation, a general defect in plasma cell differentiation, or even a reduced mitotic activity at later time points.

**Figure 2:**
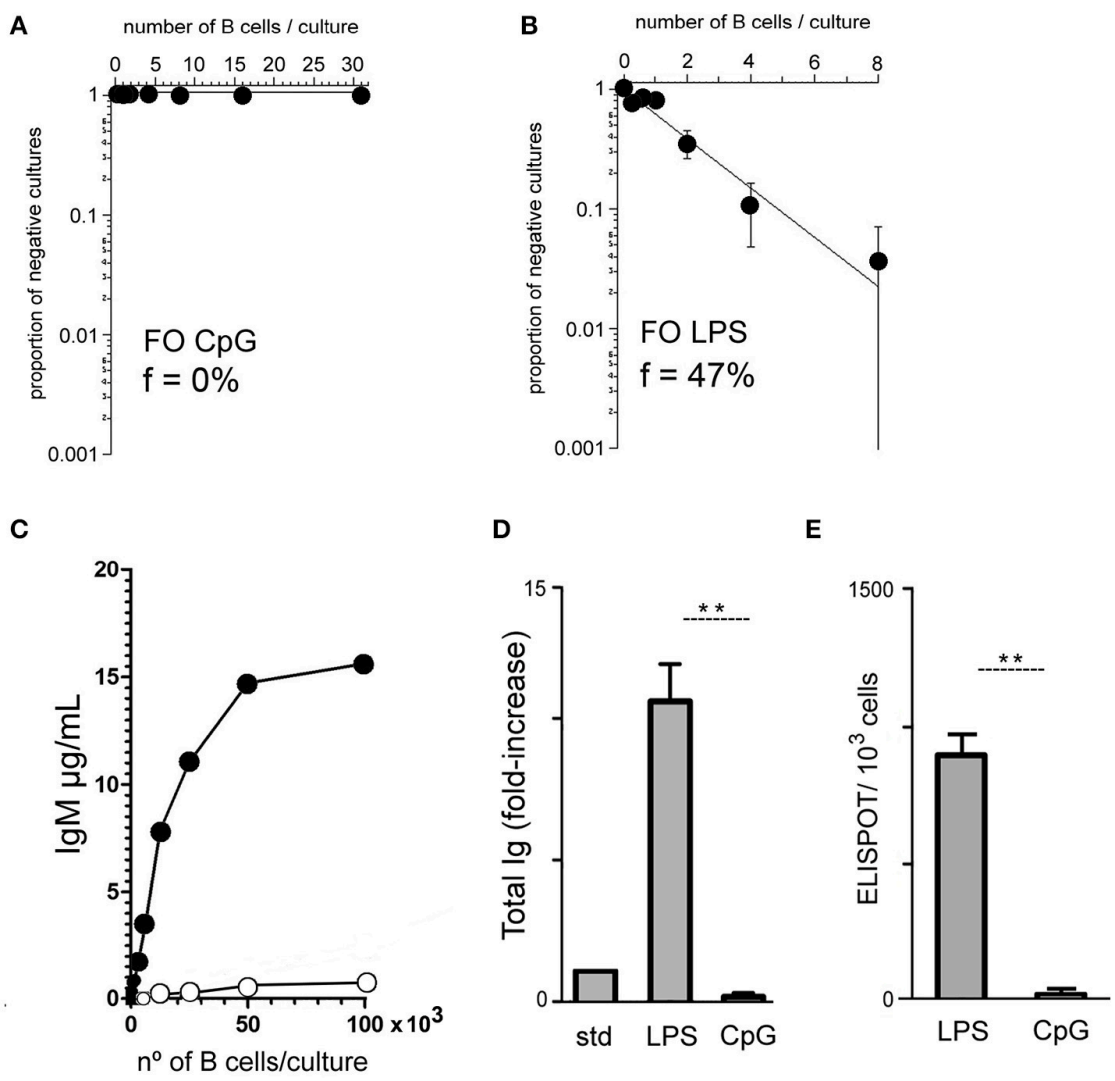
Limiting dilution analysis of B cell responses to TLR4 and TLR9 agonists. Varying numbers of purified splenic FO B cells were cultured for 7 days upon a monolayer of irradiated S17 feeder cells in the presence of CpG 1 μg/ml **(A)** or LPS 20 μg/ml **(B)**; at the end of the culture the supernatants were harvested for detection of secreted IgM and the percentage of positive/negative cultures for IgM secretion was scored and plotted according to Poisson statistics for the estimation of the frequency of responding cells. Graphs show the results of one experiment from a total of three, with similar results. **(C)** Varying numbers of purified splenic FO B cells were cultured for 7 days upon a monolayer of irradiated S17 feeder cells in the presence of CpG 1 μg/ml or LPS 20 μg/ml; at the end of the culture the supernatants were harvested for detection of secreted IgM; data show the results of one experiment; three independent experiments were done with similar results. **(D)** Purified splenic FO B cells, 1 × 10^3^ cells/well, were cultured for 7 days upon a monolayer of irradiated S17 feeder cells in the presence of LPS 20 μg/ml or CpG 1 μg/ml; at the end of the culture the supernatants were harvested for measuring of secreted total Ig by ELISA; the results are expressed as fold increase relative to a standard (STD) containing a mixture of different mouse Ig isotype (IgM+IgG+IgA) standards, 1 μg/ml each; graph shows mean ± SEM results from one experiment with triplicates cultures for each group; results were considered statistically different, Student's *t*-test if *p* < 0.01 (^**^); two independent experiments were done with similar results. **(E)** Splenic FO B cells were cultured for 96 h at cell density of 1 × 10^3^ cells/well upon a monolayer of irradiated S17 feeders cells; cultures were stimulated with LPS 20 μg/ml or CpG 1 μg/ml; at the end of the culture, cells were harvested and assayed for ELISPOT of total Ig secreting cells; ELISPOTs for LPS: 899 ± 69; ELISPOTs for CpG: 22 ± 4; graph shows mean ± SEM results from one experiment with duplicates cultures for each group; results were considered statistically different, Student's *t*-test if *p* < 0.01 (^**^); two independent experiments were done with similar results.

To approach this question in a quantitative manner, we first set up B cell cultures under limiting dilution conditions to determine the frequencies of FO B cells generating IgM-secreting cells in response to CpG or LPS stimuli (17). For that purpose, purified FO B cells were cultured on a monolayer of irradiated S17 stromal cells, following a well-established and efficient protocol for limiting dilution cultures (28). After seven days of culture, the supernatants were evaluated for the presence of secreted IgM; the percentage of positive wells was scored and the data fitted to a Poisson distribution. The frequency of sorted splenic follicular B cells (FO B) secreting IgM in response to CpG stimulus was found to be minimal, often below the threshold of the assay (<1/1,000 responding B cells) (Figure 2A); for LPS, the frequency of FO B cells secreting IgM in the experiment data shown in Figure 2B was calculated as 47 %. These results were further confirmed in two other independent experiments where sorted FO B cells responded to LPS with frequencies of 56 and 57%, and to CpG, 0.0 and 0.33% (data not shown). The results obtained in the limiting dilution cultures were thus consistent with the much lower amount of IgM measured in 7-days high-density -cultures of FO B cells, suggesting that FO B cells are indeed not undergoing plasmocyte differentiation.

We also did experiments culturing higher numbers of FO B cells, from 100 to 100,000 B cells/culture, on a monolayer of irradiated S17 stromal cells. IgM only started to be detected in the supernatant of FO B cell cultures with more than 10,000 cells/well (Figure 2C). Of note, the data obtained showed a progressive saturation of the response to LPS in higher cell-density cultures, possibly due to exhaustion of culture medium (32); this point is even more clear in cultures with total splenic B cells (Supplementary Figure 2). In order to exclude this artefactual condition, when comparing the responses to LPS and CpG, the next experiments were all done with cell-density cultures not exceeding 10,000 cells/well, avoiding high-density culture conditions, >0.5 × 10^6^ B cells/ml, to ensure the linearity of the assay and proper interpretation of the data.

We next investigated whether the lower amount of IgM measured in supernatants of CpG-stimulated FO B cells could reflect an early and massive class switch. For that, FO B cells were cultured at 1,000 cells/well, upon a monolayer of irradiated S17 stromal cells; at this cell density, FO B cells were actively proliferating in response to LPS and CpG, as verified by CFSE dilution assay (not shown) and total Ig was measured at the end of 7-days culture. The results showed that these cells did not produce detectable amounts of Ig of any isotype (Figure 2D); results were independently confirmed by ELISPOT assay (Figure 2E). Therefore we concluded that CpG is indeed unable to promote the generation of Ig secreting plasmocytes from FO B cells.

**Figure 3:**
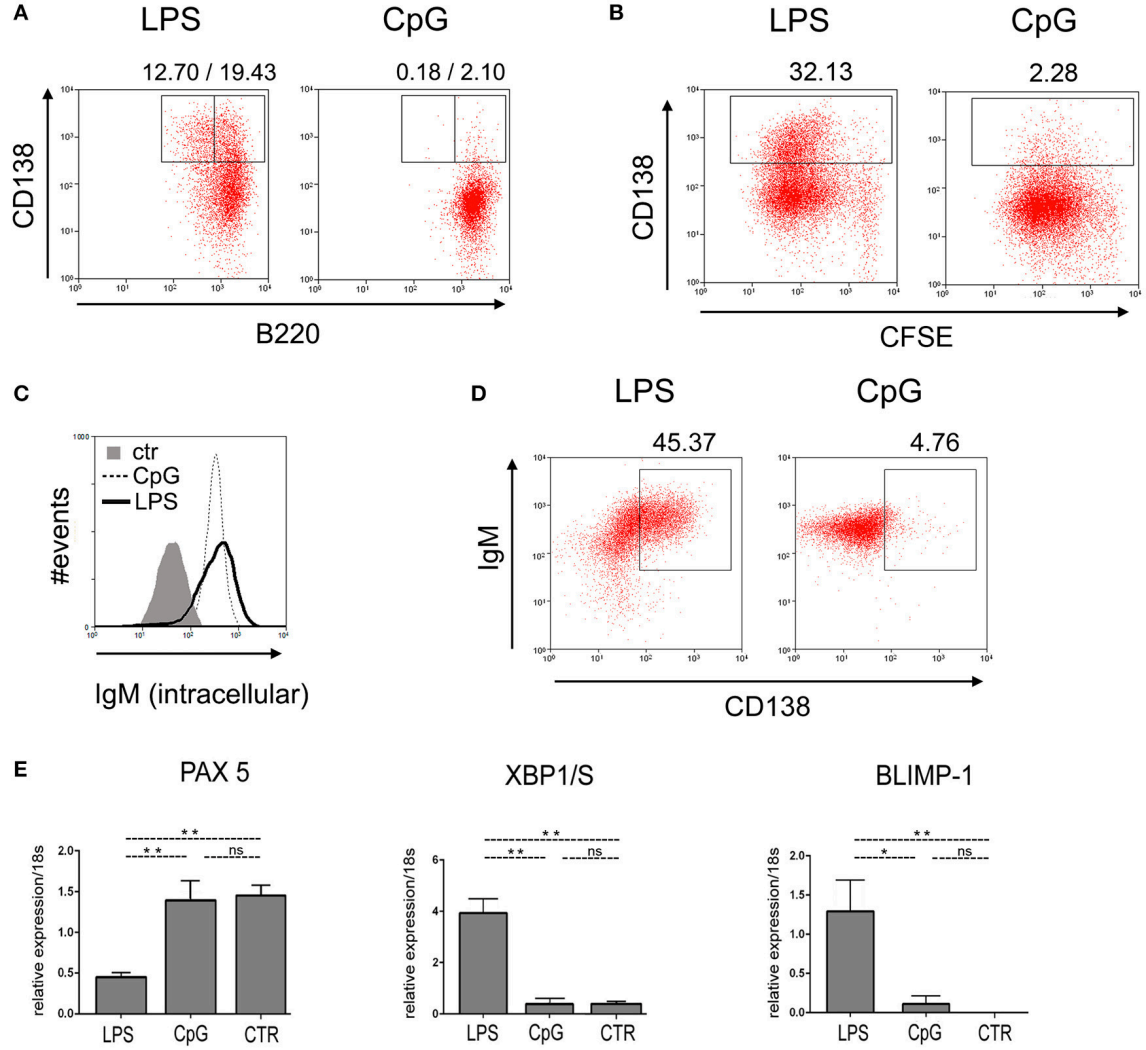
Phenotypic characterization of plasma cell differentiation induced by TLR4 and TLR9 agonists. Purified B220+ spleen B cells, 1 × 10^3^ cells/well, were loaded with CFSE and cultured upon a monolayer of irradiated S17 feeder cells and stimulated with LPS 20 μg/ml or CpG 1 μg/ml, for 96 h; cells were then harvested and **(A)** stained for the expression of B220 vs. CD138; **(B)** analyzed for numbers of mitotic divisions, by flow cytometry vs. expression of CD138. Cells were cultured as in **(A)**, and stained for the amount of intracellular IgM **(C)**; or analyzed for the expression of intracellular IgM vs. CD138 **(D)**; (**A–D)** show the results of one experiment; two independent experiments were done with similar results. **(E)** Purified splenic follicular B cells, 1 × 10^3^/well, were cultured for 96 h on a monolayer of irradiated S17 feeder cells in the presence of LPS 20 μg/ml or CpG 1 μg/ml; the expression of the messenger RNA for transcription factors PAX5 (up), XBP1/S (center) and BLIMP1 (down) were measured by qPCR; control expression of the transcription factors was measured in fresh cells (CTR), before the beginning of the culture. Data shown in **(E)** were compiled from two independent experiments; graph shows mean ± SEM results for each group; statistical comparisons between different groups (LPS, CpG, and Ctr) were done using ANOVA with Bonferroni post-test (results were considered statistically different if *p* < 0.05 (^*^); *p* < 0.01 (^**^); (ns), not significant.

To further characterize the phenotype of B cells responding to LPS and CpG stimuli, we analyzed the expression of cell surface markers and transcription factors associated with plasma cell differentiation. Cultured FO B cells were first analyzed by flow-cytometry for the cell-surface expression of B220 and CD138. Figure 3A shows that, on day 4 of culture, a significant fraction of B cells stimulated with LPS down-regulated the expression of B220 and up-regulated the expression of CD138, a classical phenotypic alteration that accompanies the generation of plasmablasts and plasma cell differentiation (21); the same was not observed in CpG-stimulated cultures (Figure 3A); expression of CD138 correlated with cell cycle division (Figure 3B). Interestingly, the amount of intracellular IgM was found to be similar in CpG-stimulated B cells, when compared to LPS (Figure 3C); intracellular IgM increases with the expression of CD138 in LPS-stimulated FO B cells, as expected; however, the same was not observed in CpG stimulated cultures, where higher expression of intracellular IgM did not correlate with levels of CD138 expression.

We also analyzed the expression of transcription factors PAX5, XBP1/S, and BLIMP-1 by quantitative RT-PCR (Figure 3D): LPS stimulated FO B cells down-regulated the expression of PAX5 and up-regulated the expression of BLIMP-1 and XBP1/S, compared to unstimulated cultures (CTR), as expected; on the contrary, CpG stimulated FO B cells maintained high levels of PAX5, and expressed much lower levels of BLIMP-1 and XBP1/S, when compared to LPS stimulated FO B cells. Taken together, these results support the notion that CpG, although a potent mitogen for B cells, does not promote plasma cell differentiation, reinforcing the conclusion that TLR9 signaling pathway is indeed unable to trigger the canonical plasma cell differentiation program in FO B cells.

**Figure 4:**
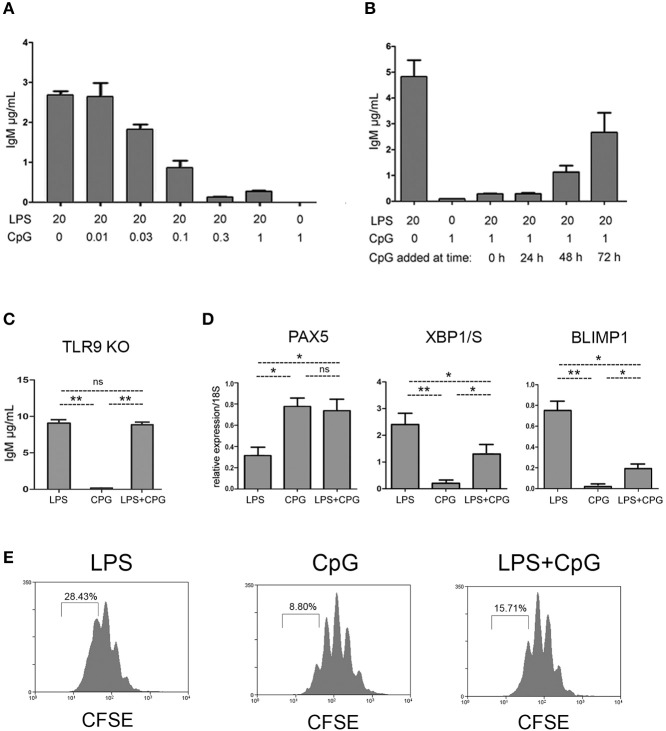
Inhibition of LPS induced plasma cell differentiation by CpG. **(A)** Splenic FO B cells, 1 × 10^3^/well, were cultured for 7 days upon a monolayer of irradiated S17 feeder cells in the presence of LPS and different doses of CpG; at the end of the culture, the supernatants were harvested and the amount of secreted IgM was measured by ELISA; graph shows mean ± SEM results from one experiment with triplicates cultures for each group; two independent experiments were done with similar results. **(B)** Splenic FO B cells, 1 × 10^3^/well, were cultured for 7 days upon a monolayer of irradiated S17 feeder cells and stimulated with LPS 20 μg/ml; 1 μg/ml of CpG was added at different time points; at the end of the culture, the supernatants were harvested and the amount of secreted IgM was measured by ELISA; graph shows mean ± SEM results from one experiment with triplicates cultures for each group; two independent experiments were done with similar results. **(C)** Splenic follicular B cells from TLR9 –/– mice, 3 × 10^3^/well, were cultured for 7 days upon a monolayer of irradiated S17 feeder cells in the presence of LPS 20 μg/ml, CpG 1 μg/ml, or LPS+CpG; IgM secretion in the supernatants were measured by ELISA; graph shows mean ± SEM results from one experiment with triplicates cultures for each group; statistical comparisons between different groups (LPS, CpG and LPS+CpG) were done using ANOVA with Bonferroni post-test (results were considered statistically different if *p* < 0.05 (^*^); *p* < 0.01 (^**^); (ns), not significant. Two independent experiments were done with similar results. **(D)** Splenic FO B cells, 1 × 10^3^/well, were cultured for 4 days on a monolayer of irradiated S17 feeder cells in the presence of LPS 20 μg/ml, CpG 1 μg/ml, or both LPS+CpG; the expression of the messenger RNA for transcription factors PAX5 (left), XBP1/S (center), and BLIMP-1 (right) was evaluated by qPCR.; Data shown in **(D)** were compiled from two independent experiments; graph shows mean ± SEM results for each group; statistical comparisons between different groups (LPS, CpG and LPS+CpG) were done using ANOVA with Bonferroni post-test (results were considered statistically different if *p* < 0.05 (^*^); *p* < 0.01 (^**^); (ns), not significant. **(E)** Splenic FO B cells, 1 × 10^3^/well, were loaded with CFSE and cultured for 4 days on a monolayer of irradiated S17 feeder cells in the presence of LPS 20 μg/ml, CpG 1 μg/ml, or both LPS+CpG; histogram shows mitotic cycles profiles measured by CFSE dilution. Histograms show the result of one experiment; two independent experiments were done with similar results.

We next investigated if the impaired plasma cell differentiation associated with CpG stimulus could be overcome by the concomitant stimulation with LPS. Strikingly, we found that plasma cell differentiation induced by LPS was severely blocked in the presence of CpG, in a dose dependent manner (Figure 4A). The inhibitory action of CpG was still fully effective when CpG was added 24 h after the beginning of cell culture, and very significant even at 48 h (Figure 4B). The inhibitory effect in response to CpG, on plasma cell differentiation induced by LPS, was totally dependent on the presence of TLR9, as demonstrated by the experiments with *Tlr9*^−/−^mice (Figure 4C). We then measured the expression of mRNA for transcription factors PAX5, XBP1/S, and BLIMP-1 in FO B cells stimulated with LPS, CpG, or LPS+CpG: BLIMP-1 expression was significantly reduced in cells stimulated with LPS+CpG, when compared to cells stimulated with LPS only (Figure 4D). The expression of XBPS/1 was partially reduced and the expression PAX5 mRNA was not diminished in cultures stimulated with LPS+CpG (Figure 4D). Cell cycle profiles were also compared for cultures stimulated with LPS, CpG or LPS+CpG (Figure 4E). The augmented number of mitotic cycles observed for LPS stimulated FO B cells, was not observed in CpG, as previously shown; LPS+CpG stimulated cultures showed an intermediate phenotype between LPS and CpG profiles, more akin to CpG. It is important to note that the number of mitotic cycles undergone by FO B cells in cultures stimulated with CpG, or LPS+CpG, is compatible with plasmablast differentiation, as shown in LPS-stimulated cultures (Figures 3B, 4E).

**Figure 5:**
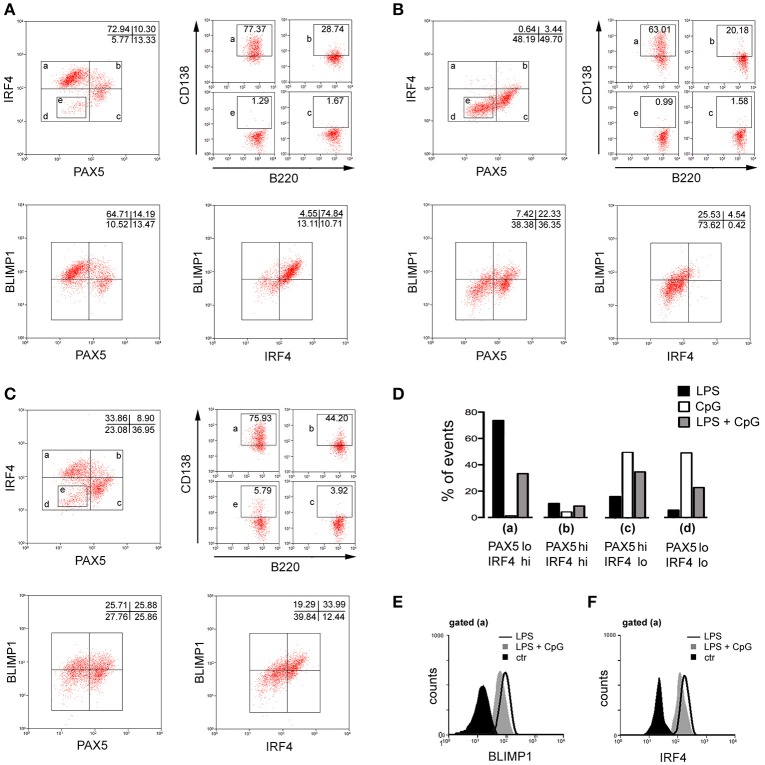
Phenotypic characterization by flow cytometry of plasma cell differentiation induced by LPS, CpG, and LPS+CpG. Purified splenic follicular B cells, 5 × 10^3^/well, were cultured for 96 h upon a monolayer of irradiated S17 feeder cells in the presence of LPS 20 μg/ml or CpG 1 μg/ml; many replicates of each culture were then harvested, pooled, and stained for the expression of B220, CD138, PAX5, BLIMP1, IRF4 and analyzed by flow cytometry. Three thousands events are shown for [PAX5 x IRF4], [PAX5 x BLIMP1] and [BLIMP1 x IRF4] dot plots. [PAX5 x IRF4] dot plot was subdivided into distinct regions, (a), (b), (c), and (e), and the expression of B220 x CD138 is shown for events gated on these regions, 1,000 events/region. **(A)** LPS, **(B)** CpG, **(C)** LPS+CpG. **(D)** Histogram bars summarizing the data shown in [PAX5 x IRF4] dot plots in **(A–C)**. **(E)** Histogram of BLIMP1 expression in cells cultured in different conditions, gated for region (a). **(F)** Histogram of IRF4 expression in cells cultured in different conditions, gated region (a). Data shown in figure are representative of two independent experiments, with similar results.

Expression of transcription factors at the protein level was also assayed by flow cytometry (Figure 5). As expected, LPS-stimulated B cells up-regulated BLIMP1, IRF4, while down-modulating PAX5 (Figure 5A). The dot plot (PAX5 vs. IRF4) was divided into four regions (a), (b), (c), and (d), corresponding to cells in different stages of PBs/PCS differentiation: (a) PAX5-lo/IRF4–hi, corresponds to advanced PBs/PCs with higher expression of CD138, down-modulation of B220 and up-regulation of BLIMP1; (b) PAX5-hi/IRF4–hi, corresponds to less advanced PBs/PCs with moderately increased expression of CD138 and BLIMP1; (c) PAX5-hi/IRF4-lo, corresponds to B cells with little expression of CD138 and BLIMP1; (e) a minor PAX5^lo^/IRF4^lo^ population of unknown significance, indicated by a rectangular gate within the region (d). In cultures stimulated with CpG, expression of BLIMP-1 was significantly diminished at the protein level and IRF4 was not up-regulated at all (Figure 5B). Interestingly, a significant population down-modulated PAX5 protein expression in CpG stimulated cultures, but IRF4 expression was not induced in those cells; curiously, these cells displayed the unusual PAX5^lo^/IRF4^lo^ phenotype (e), mostly absent in LPS cultures. In cultures stimulated with LPS+CpG, a significant population of cells expresses intermediate levels of BLIMP-1 and IRF4, with concomitant down-modulation of PAX5 (Figure 5C). Of note, in all culture conditions, cells with phenotypes (a), (b), (c), and (e), maintained their characteristic expression of CD138. The percentages of cells displaying these phenotypes are grouped and showed in Figure 5D. Figures 5E,F show the histograms of BLIMP1 and IRF4 expressions, in cells with phenotype (a), in all culture conditions.

**Figure 6:**
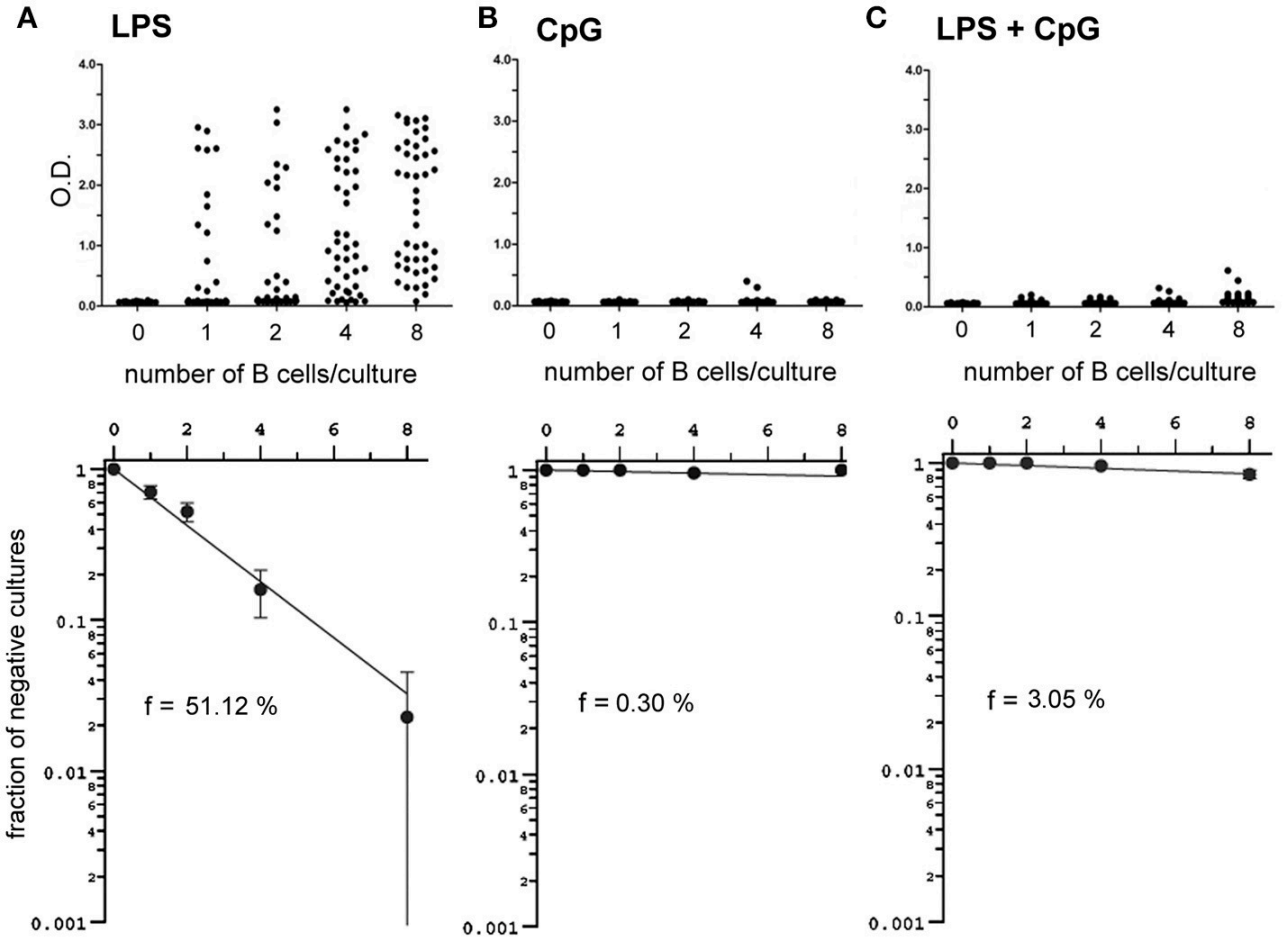
Frequencies and magnitude of clonal responses and of B cell cultures responding to LPS, CpG and LPS+CpG under limiting dilution conditions. Varying numbers of splenic FO B cells, 1 to 8 cells/well, were cultured for 7 days upon a monolayer of irradiated S17 feeder cells in the presence of LPS 20 μg/ml **(A)**, CpG 1 μg/ml **(B)**, or LPS+CpG **(C)**; at the end of the culture the supernatants were harvested for detection of secreted IgM by ELISA; the magnitude of the OD value scored for supernatants are shown for each individual culture (upper panels); 44 replicates were done for each cell number/well; Poisson statistics (lower panels) was used for the estimation of the frequency of responding cells. Data shown in figure are representative of two independent experiments, with similar results.

The inhibition of LPS-induced plasma cell differentiation by CpG was further confirmed at the single cell level, with limiting dilution cultures of FO B cells stimulated either with LPS, CpG, or LPS+CpG (Figure 6). As previously observed, CpG alone was unable to generate positive cultures for the production of IgM; the addition of CpG to LPS resulted in a marked reduction of the frequency of B cells responding to LPS, from 51 to 3% (Figure 6A,C); importantly, B cell cultures considered as positive under LPS+CpG stimuli produced very little IgM when compared to LPS-only stimulated FO B cells, indicating that the plasmocyte differentiation by LPS was impaired by the addition of CpG, even in positive-scoring cultures.

## Discussion

B1a and MZ B cells respond better to TLR stimuli than FO B cells, in kinetics and magnitude (22–25). However, for LPS stimulus, FO B cell differentiation into PBs/PCs is only delayed when compared to MZ B cells and their response is also massive, both in frequency of PB generation and magnitude of Ig secretion (22, 23). In limiting dilution assays, a vast majority of splenic B cells from C57BL/6 mice generate PBs/PCs, thus showing that TLR4 signaling is able to fully promote plasmocyte differentiation of FO B cells (28). B cell responses, comparing TLR4 and TLR9 agonists, have been studied by different groups; their results indicate that signaling by both TLRs induce the generation of Ig-secreting cells from FO and MZ B cells in high-density culture condition, > 50,000 B cells/96-microplate well (24, 25, 33, 34). The view supported by these studies propagates the notion that both stimuli are able promote polyclonal B cell proliferation and PB/PC differentiation of FO and MZ B cells, however with different efficiencies. Whether the reduced amount of PB generation in FO B cell cultures is due to delayed kinetics, to a general defect in PB differentiation or to a lower frequency of fully responding FO B cells, was not investigated.

The clear-cut results we obtained here with limiting dilution assays, quantifying the generation of Ig-secreting B cells in response to TLR9 stimulus, do not align with the above current view. We did a total of 6 LDA experiments with purified FO B cells, comparing LPS and CpG stimuli: (i) with LPS, around 50% responded, generating colonies of PBs/PCs that secreted significant amounts of IgM per growing clone (3–30 ng/ml) after 7 days of culture; (ii) in contrast, the vast majority of CpG-stimulated LDA cultures scored negative for IgM, below the minimum threshold of detection (0.6 ng/ml). Summing up the series of LDA experiments we performed here, comparing the responses of LPS and CpG, no less than 10,000 FO B cells were screened for response to CpG by Ig secretion, almost all with negative results, indicating a frequency of immunoglobulin-secreting colonies certainly below 1/1,000. For comparison, in our hands, CpG stimulated MZ B cells generate IgM secreting clones with a frequency around 20 % (not shown).

The absence of IgM, or any other isotype in the supernatants of LDA in low density cultures (1,000 cells/well) of CpG-stimulated B cells, could suggest the lack of some factor that could be present in high-density culture condition. However, we think this is unlikely because S17 stromal cells have been described as optimal for LDA B cell assay with LPS and for B cell precursor growth and maturation in the absence of exogenous stimuli (28, 35, 36). Also, the production of a hypothetical inhibitory factor by S17 in the presence of CpG can be ruled out because CpG inhibited Ig secretion in LPS-stimulated B cells in a TLR9 dependent manner; if such a factor would be produced by S17, it should have inhibited plasmocyte differentiation by LPS irrespective of TLR9 expression on B cells. We conclude that signaling through TLR9 is indeed unable to induce plasma cell differentiation. We have added different cytokines (IL-2, IL-4, IL-6, IL-21, and BAFF) and ligands (anti-CD40 agonist mAb) to CpG cultures, but none could induce the appearance of Ig secreting cells (not shown).

Our results indicate that CpG-stimulated FO B cells, although proliferating vigorously, do not differentiate into plasma cells (Figure 2). To our knowledge, this is the first description that TLR9 signaling is totally unable to promote FO B cell plasmocyte differentiation. This conclusion finds support in the comparative analysis of the expression profiles of transcription factors involved in PBs/PCs differentiation program in CpG- and LPS-stimulated B cell cultures. In agreement with published data, we found that B cells, in LPS stimulated cultures, up-regulate the expression of IRF4, BLIMP1, XBP1/s, and down-regulate PAX5 (37–42). In contrast, in CpG-stimulated cultures, B cells do not up-regulate IRF4, BLIMP1 expression is diminished and XBP1/s is not induced. Other classical phenotypic alterations associated with PC differentiation program are also absent in CpG-stimulated cultures: CD138 expression was not increased and B220 down-modulation was not observed.

We would like to point out that some of the phenotypic characteristics we observed here in CpG-stimulated FO B cells agree with data presented in previous studies (24, 34), but none of these studies demonstrate the incapacity of TLR9 signaling to promote PB/PC differentiation of FO B cells. On the contrary, in one of these studies, the relative magnitudes of FO B cells responses to LPS and CpG seems to suggest a similar efficiency of both agonists in high-density culture conditions (24). Here, on the other hand, a clear-cut difference was revealed between TLR4 and TLR9 concerning FO B cell plasmocyte differentiation. The reasons for these discordant results may be explained by the different culture conditions, low-density cultures vs. high-density cultures, and different doses of LPS. In high-density cultures it cannot be ascertained how much of the secretion of Ig may originate from cell contaminants; for instance, 0.5 % of MZ or B-1 contaminants among 100,000 cells/well adds up to 500 cells/well, that could multiply into 16,000 after 5 mitosis only; this is a critical point that, in our opinion, has not received enough attention. Moreover, the quantitative comparison based on the enumeration of ELISPOT in high-density cultures at early time points is also difficult to interpret because only a minority of cells is secretory in those cultures and may not represent the behavior of the cell population as a whole. Contrasting with this approach, it was established that all LPS-growing clones in LDA become secretory, when assayed on days 4, 5, and 6 (17). Here, the data we obtained in low density cultures, 1,000 cells/well, on S17 monolayer of feeder cells, agree with these classical studies within the margin of error: 1,000 SPOTS out of 1,000 plated cells were counted on day 4, the whole population being represented in the assay.

Alternatively, the difference between results obtained with low-density cultures vs. high-density cultures could also be explained if a very limited amount of Ig secretion, bearing no correlation with PB differentiation, is induced by TLR9 signaling. If that would be the case, a barely detectable Ig production to CpG stimuli in low-density culture may become significant by increasing cell number/culture, thus augmenting TLR9-dependent FO B cell response; if this is the case, it will also appears to increase in respect to LPS stimulated cultures, because of saturation of PB generation of the latter at higher cell density (Supplementary Figure 2) (32). Whatever the case, the data showed here in low-density and LDA cultures, clearly indicates that TLR9 signaling does not induce the canonical PB differentiation of FO B cells

CpG-stimulated B cells express lower levels of BLIMP1 (Figures 3, 5); interestingly, their phenotype bears some similarities with BLIMP1-deficient B cells (43, 44). BLIMP1-deficient B cells also do not up-regulate IRF4 and secrete minimal amounts of Ig; they do not produce the secretory form and accumulate intracellular Ig, as observed here for CpG-stimulated B cells (Figure 3B). BLIMP1 expression has been shown to be crucial for the proper generation of PBs/PCs, which respond to graded levels of BLIMP1 (43–45); a lower expression of BLIMP1 and IRF4 in CpG-stimulated B cells may not sustain XBP1/s expression (46, 47), arresting B cell differentiation at a pre-plasmablast stage, as previously suggested (40, 43). It is interesting to speculate whether the cells showing the IRF4^lo^/PAX5^lo^ phenotype, which are abundant in CpG-stimulated cultures, but rare in LPS-, may represent an alternative fate of B cell differentiation under TLR9 stimuli.

More surprising was the suppression, by CpG, of plasmocyte differentiation induced by LPS (Figure 4A); this inhibition depended on the expression of TLR9 by B cells (Figure 4C). It was established long ago that BCR ligation inhibits the generation of PBs/PCs in B cells responding to LPS (32). This seems to be an early decision; that is, following BCR ligation, LPS-activated B cells are either fully inhibited or not, the alternative being established early after activation. While inhibition of PC differentiation by BCR engagement is only active in the first 6 h, inhibition by CpG is maximal even if added 24 h after LPS stimulus (Figure 4B), indicating a distinct intracellular mechanism. Crosstalk between BCR and TLR4 signaling pathways, mediated by ERK, has been characterized in the inhibition of BLIMP1 expression (48, 49); here BLIMP1 expression was found to be diminished in TLR9 inhibited PB/PC differentiation, but we could not identify a similar mechanism (not shown).

The finding that TLR9 signaling antagonizes LPS-induced plasmocyte differentiation was unexpected, as TLR4 and TLR9 signaling pathways share many intracellular mediators (50). Here we have not attempted to investigate the mechanisms leading to the diminished expression of transcription factors in B cells stimulated with both agonists (Figure 5); further studies focused on intracellular pathways are necessary to unveil what is altered and how. It is interesting to note that the presence of the IRF4^lo^/PAX5^lo^ population in CpG and LPS+CpG stimulated cultures may suggest that TLR9 inhibition is acting downstream the events coordinating the down-modulation of PAX5; as such, the action of TLR9 signaling, inhibiting the expression of IRF4, BLIMP1 and XBP1/s seems to overrule the release of negative control of PAX5 upon these genes. Of note, Ig secretion in LPS+CpG FO B cell cultures was profoundly inhibited although the expression of canonical transcription factors was only partially affected, suggesting the involvement of other critical factors controlling PB differentiation.

The results described here disclose a fundamental difference between TLR4 and TLR9 stimuli concerning plasmocyte differentiation and support the conclusion that TLR9 signaling does not promote PB/PC differentiation of FO B cells. This conclusion may have important consequences for the conceptual scheme that suggests the breaking of tolerance to nuclear antigens by the engagement of TLR9 following BCR-mediated endocytosis of those antigens associated with DNA (27). On one hand, our data suggest that additional signals may be necessary to generate plasmocytes secreting self-reactive antibodies under these circumstances. On the other hand, we may speculate that the inhibitory effects of TLR9 signaling in plasmocyte differentiation could be inversely related to the control of autoantibody production by TLRs: failure of this pathway may favor the appearance of self-reactive secretory plasmocytes. This scenario, if confirmed *in vivo*, would enlarge the current perspective on the role of TLR9 in autoimmunity. This interpretation aligns with surprising results showing that absence of TLR9 signaling in B cells actually enhanced autoantibody production (51).

Keeping with the central postulates of Clonal Selection, antibody responses occur through antigen-dependent selection of rare clones with the appropriate specificity, followed by proliferative expansion of the selected cells and their terminal differentiation to high-rate antibody secretion plasma cells. Interestingly, however, these two processes are mutually excluded, as plasma cell differentiation is “terminal” and incompatible with maintained proliferation. It follows that early PC differentiation of activated cells severely limits clonal expansion, maximal levels of circulating antibody, and the establishment of “memory” and affinity maturation; in contrast, investing in proliferation for too long, would deprive the organism of the essential antibodies it needs to fight the initial phases of infection, for example (52). It is not surprising, therefore, that the balance between proliferation and terminal differentiation is highly regulated by the nominal antigen itself through the surface BCR (28). At face value, the opposing effects of TLR9 in plasma cell differentiation are not antigen-specific; however, if capture of DNA-associated nuclear antigens by BCR is the preferred mechanism to engage TLR9, it will place TLR9 inhibition of under the control of the BCR specificity, because only anti-nuclear antigens clones would then be suppressed, reconciling this interpretation with the classical theoretical paradigm.

In conclusion, the findings described here involving TLR9 signaling in FO B cells modify our current conceptual view of the action of this receptor; it opens a new perspective on the potential role of TLR9 in autoimmunity and have implications for the balance between proliferation and terminal differentiation of B lymphocytes, adding a new layer of complexity in the regulation of clonal sizes of B cell repertoires engaged in humoral immune responses.

## Author contributions

BB, AG, FC, FM, AV, LT, HdMG, and AN participated in intellectual and experimental work. AC and MB participated in intellectual work. AN wrote the paper.

### Conflict of interest statement

The authors declare that the research was conducted in the absence of any commercial or financial relationships that could be construed as a potential conflict of interest.

## Supplementary material

Supplemmentary Figure 1 FO B cells sorting gates. Single cell suspensions of splenic cells were prepared as described in Material and Methods and stained with mabs against B220, CD93, CD23, CD21. Sorting of FO B cells was done by first gating the lymphoid population on FCS/SSC dot plot, then gating on B220+CD93—cells and finally on CD21+CD23+ narrow gate within the core of the FO B cell population. Purity of the sorted population was ascertained comparing pre-sorting and pos-sorting B cells using a gate surrounding the whole FO B cell population.

Supplemmentary Figure 2 Influence of cell density on the saturation of plasmocyte generation *in vitro*. Varying numbers of total splenic B cells were cultured for 7 days upon a monolayer of irradiated S17 feeder cells in the presence of CpG 1 μg/ml or LPS 20 μg/ml; at the end of the culture the supernatants were harvested for detection of secreted IgM.
