# Supplementary figures and images for: TLR9 Signaling Suppresses the Canonical Plasma Cell Differentiation Program in Follicular B Cells
Source: Front Immunol. 2018 Nov 28;9:2281. doi: 10.3389/fimmu.2018.02281 (PMC6279956; doi:10.3389/fimmu.2018.02281)

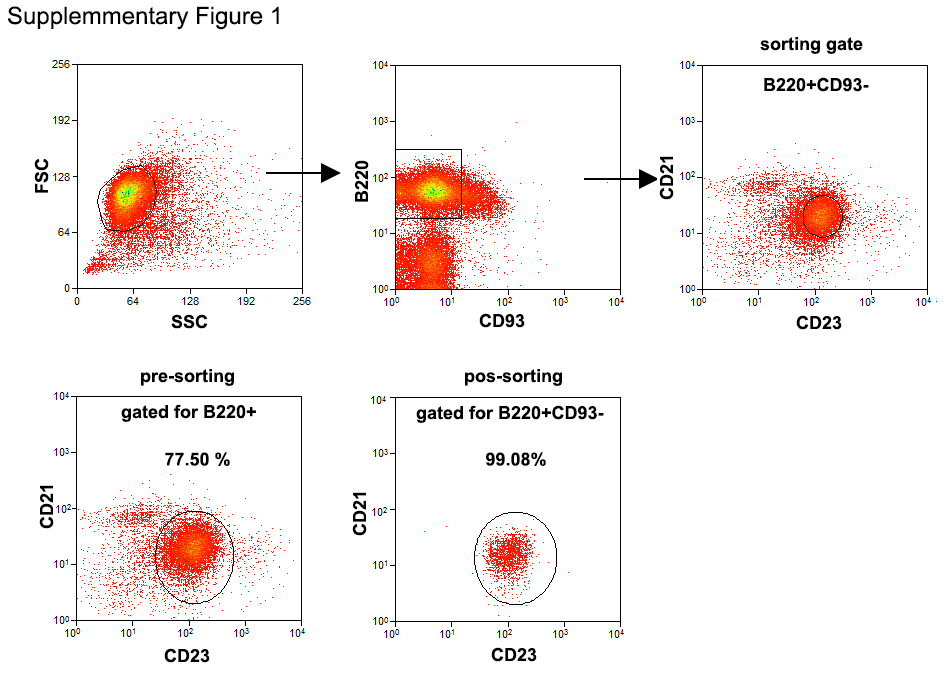

Supplement: Supplemmentary Figure 1 — FO B cells sorting gates. Single cell suspensions of splenic cells were prepared as described in Material and Methods and stained with mabs against B220, CD93, CD23, CD21. Sorting of FO B cells was done by first gating the lymphoid population on FCS/SSC dot plot, then gating on B220+CD93—cells and finally on CD21+CD23+ narrow gate within the core of the FO B cell population. Purity of the sorted population was ascertained comparing pre-sorting and pos-sorting B cells using a gate surrounding the whole FO B cell population. [file Image_1.TIF]

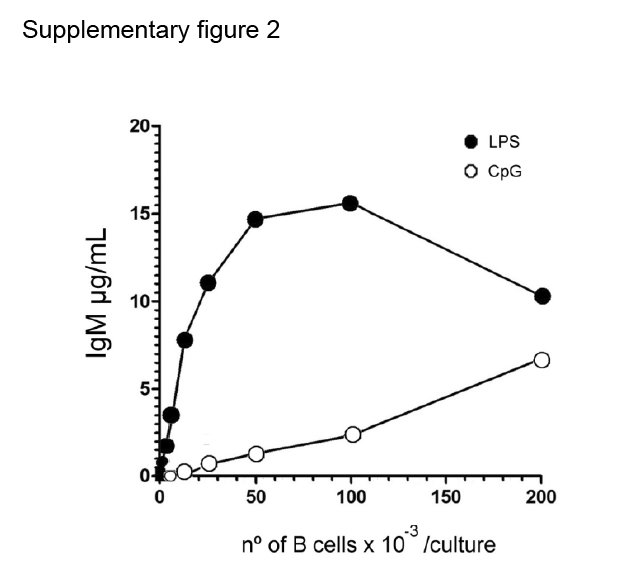

Supplement: Supplemmentary Figure 2 — Influence of cell density on the saturation of plasmocyte generation in vitro. Varying numbers of total splenic B cells were cultured for 7 days upon a monolayer of irradiated S17 feeder cells in the presence of CpG 1 μg/ml or LPS 20 μg/ml; at the end of the culture the supernatants were harvested for detection of secreted IgM. [file Image_2.TIF]
